# Radical scavenging capacity, antibacterial activity, and quantum chemical aspects of the spectrophotometrically investigated iridium (III) complex with benzopyran derivative

**DOI:** 10.3389/fphar.2022.945323

**Published:** 2022-09-02

**Authors:** Masrat Mohmad, Nivedita Agnihotri, Vikas Kumar, Mohammad Azam, Saikh Mohammad Wabaidur, Raj Kamal, Rakesh Kumar, Mahboob Alam, Sadegh Kaviani

**Affiliations:** ^1^ Department of Chemistry, Maharishi Markandeshwar (Deemed to Be University), Mullana, Ambala, India; ^2^ Department of Biotechnology, Maharishi Markandeshwar (Deemed to Be University), Mullana, Ambala, India; ^3^ Department of Chemistry, College of Sciences, King Saud University, Riyadh, Saudi Arabia; ^4^ Department of Chemistry Kurukshetra University, Kurukshetra, India; ^5^ Department of Chemistry, MCM DAV College, Kangra, Himachal Pradesh, India; ^6^ Department of Safety Engineering, Dongguk University, Gyeongju, South Korea; ^7^ Department of Physics, Kazan Federal University, Kazan, Russia

**Keywords:** iridium, quantum chemical studies, spectrophotometric determination, antibacterial, antioxidant

## Abstract

A comprehensive aqueous phase spectrophotometric study concerning the trace level determination of iridium (III) by its reaction with benzopyran-derived chromogenic reagent, 6-chloro-3-hydroxy-7-methyl-2-(2′-thienyl)-4-oxo-4*H-*1-benzopyran (CHMTB), is performed. The complexing reagent instantly forms a yellow complex with Ir (III) at pH 4.63, where metal is bound to the ligand in a ratio of 1:2 as deduced by Job’s continuous variations, mole ratio, and equilibrium shift methods. The complex absorbs maximally at 413–420 nm retaining its stability for up to 4 days. An optimum set of conditions have been set with respect to the parameters governing the formation of the complex. Under the set optimal conditions, the Ir (III)-CHMTB complex coheres to Beer’s law between 0.0 and 1.5 µg Ir (III) mL^−1^. The attenuation coefficient and Sandell’s sensitivity are, respectively, 1.18×10^5^ L mol^−1^ cm^−1^ and 0.00162 μg cm^−2^ at 415 nm. The correlation coefficient (r) and standard deviation (SD) were 0.9999 and ± 0.001095, respectively, whereas the detection limit as analyzed was 0.007437 μg ml^−1^. The interference with respect to analytically important cations and complexing agents has been studied thoroughly. It is found that the majority of the ions/agents do not intervene with the formation of the complex, thus adding to the versatility of the method. The results obtained from the aforesaid studies indicate a simple, fast, convenient, sensitive, and versatile method for microgram analysis of iridium (III) using CHMTB as a binding ligand. Furthermore, the studied complex is subjected to the evaluation of antibacterial and antioxidant capacity by employing the Agar Diffusion assay and DPPH^.^ radical scavenging method, respectively. The results obtained from the mentioned assays reveal that the investigated complex possesses significant potency as an antibacterial and antioxidant agent. Finally, the computational approach through DFT of the formed complex confirmed the associated electronic properties of the studied complex.

## Introduction

Owing to the multiplex electronic and stearic properties and, therefore, aptness to undergo ligand exchange with biomolecules, group 9 transition metals and their complexes have attracted considerable attention from inorganic chemists and are thus widely explored (Lu et al., 2015). Iridium is one of the most important rare elements found in the earth’s crust and normally exists in uncombined form or natural alloys such as osmiridium (osmium rich) and iridosmine (iridium rich). Surprisingly, despite its lower reactivity, iridium forms a plethora of compounds with oxidation states ranging from −3 to +9. Iridium, in addition to its unique electronic arrangement, has minimal toxicity and environmental impact, making it a green element. Due to their unique characteristics, iridium and its complexes have a wide range of applications, including industrial, medical, and catalysis (Singh, 2016). Besides this, iridium and its complexes are used in electrical and electrochemical operations, in energy production, as anticancer agents, and for the fixation and conversion of atmospheric carbon dioxide into useful products (Singh, 2016).

Thus, there is an increasing demand for the development of analytical procedures, preferably simple, sensitive, and selective ones to broaden the horizon of the applicability of iridium and its complexes. Sophisticated techniques such as NAA (Miura et al., 2020), spectrofluorimetric (Druskovic et al., 2004), AAS (Ingo et al., 2008), voltammetry (Locatelli, 2013), atomic emission and flame emission spectrometry (Jackson and Qiao, 1992), ICP-OES (Zhang and Tian, 2015), ICP-MS (Yi and Masuda, 1996), sequential flow injection analysis (Khomutova et al., 2013), imprinted polymer-based method (Sid Kalal et al., 2013), and individual catalytic method (Kawamura et al., 2011) have been employed for the purpose. However, the wider application of these techniques is restricted by several factors such as instrumentation costs, technical know-how, and maintenance issues. In order to combat these flaws, a wide range of versatile spectrophotometric methods have been used for the trace level determination of iridium in its complexes (Druskovic and Vojkovic, 2003; Li-Ming et al., 2008; Kuchekar et al., 2014; Borgave and Barhate, 2016; Kuchekar et al., 2019). To date, many spectrophotometric methods, both extractive and non-extractive, have been proposed to study iridium complexes; the aqueous phase non-extractive study, however, is more effective than the extractive ones as the latter involve cumbersome procedures.

The current method elucidates the non-extractive aqueous phase spectrophotometric determination of Ir (III) as its coordination complex with 6-chloro-3-hydroxy-7-methyl-2-(2′-thienyl)-4-oxo-4*H-*1-benzopyran (CHMTB). A keen literature survey reveals that CHMTB, one of the benzopyran derivatives, has so far not been used for the analytical determination of Ir (III). Furthermore, because the suggested approach does not use solvent extraction, the use of organic solvents that have been known to be harmful, polluting, and carcinogenic are avoided, keeping in view the therapeutic properties of iridium complexes (Mukherjee et al., 2014; Lapasam et al., 2020; Mohmad et al., 2022). Ir (III)-CHMTB was investigated for antibacterial and antioxidant properties by following the standard protocols and was found to exhibit the same. Moreover, the complex was found to be a more potent antibacterial and antioxidant agent, as was inferred from MIC and IC_50_ values, respectively. Insight and comprehension into the electronic structural details and chemical reactivity of the complex were accomplished using DFT, which further reinforced the basis of structural properties of the studied complex. This conforms with the computational studies as already been performed for the analogous metal complexes (Kataria et al., 2017; Dhonchak et al., 2021; Mohmad et al., 2022).

## Experimental

### Instrumentation

All the absorbance measurements were performed using UV-Vis spectrophotometer (double beam; EI-2375) with 1 cm identical quartz cuvettes. Highly precise electronic weighing balance (EI-101) and sensitive pH meter (alpha-01) with combination electrodes were used, respectively, for quantifying weights and pH observations. An exhaustive contamination control was followed while carrying out the experiments. The calibrated glassware used during the experiments was cleaned by soaking it in dilute HCl for 12 h followed by rinsing with double-distilled water.

### Reagents and solutions

High purity chemicals of AR grade and deionized water were used during the test. A 100 ml of the stock solution of iridium with a strength of 1 mg Ir (III) mL^−1^ was prepared by dissolving a precisely weighed amount (0.155 g) of iridium trichloride (IrCl_3_) in 6M HCl. Working solutions of the required concentration (100 and 10 μg ml^−1^) were prepared by appropriate dilution of the stock solution with doubly distilled water. For the regulation of the ambient pH of the reaction medium, 0.5 M H_3_PO_4_ was prepared.

### Ligand solution

**FIGURE 1:**
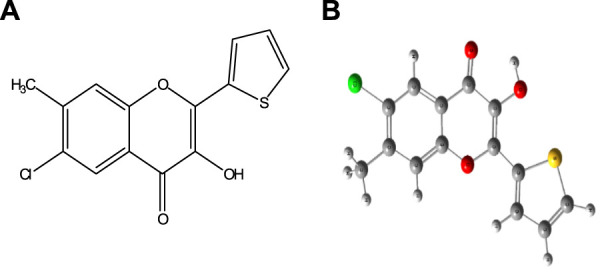
Structure of 6-chloro-3-hydroxy-7-methyl-2-(2′-thienyl)-4-oxo-4*H-*1-benzopyran (CHMTB): **(A)** 2D structure and **(B)** optimized structure.

The chromogenic reagent, CHMTB, containing two functional sites, the carbonyl group and the hydroxyl group (Figure 1), was synthesized by applying the methodology as followed in the Algar–Flynn–Oyamada (AFO) reaction (Algar and Flynn, 1934; Oyamada, 1934). 0.1% (w/v) solution of CHMTB was made by dissolving 0.025 g of it in a small amount of ethanol, followed by dilution with the same solvent up to the mark in a 25 ml graduated flask.

### Other required solutions

In order to study the diverse effects caused by most of the analytically important cations and complexing agents, required solutions were prepared from the salts by dissolving in deionized water or the mineral acids.

### Suggested procedure for determination

In order to achieve accurate and flawless results, the spectrophotometric inspection was carried out in the most optimized manner. By diluting the stock solution with appropriate amounts of double-deionized water, a working solution (10 μg ml^−1^) of the metal ion under study was prepared for analysis. For the process of complexation, 1 ml of the working solution was pipetted out in a 10 ml standard volumetric flask, 1 ml of the 0.1% reagent CHMTB solution was added to it to carry out complexation in the presence of 0.3 ml of 0.5 M H_3_PO_4_, thereby, adjusting the ambient pH 4.63 of the reaction medium. The remaining volume was made by adding double-distilled water up to the mark. After adding 1 ml of ethanolic solution of 0.1% (w/v) CHMTB, a yellow-colored complex was formed instantaneously. The optical density of the resulting complex at 415 nm was compared to that of a similarly treated reagent blank for absorbance studies. The precise amount of metal ion was calculated using the calibration curve constructed between absorbance and varying iridium concentrations (III).

### Computational details

Quantum chemistry (DFT) computations producing molecular configurations of the minimum energies molecular orbitals (HOMO-LUMO) and other properties were performed using the Gaussian 09 (Revision B.04) package. The geometry optimization was done when the system was gaseous. Gauss View 05 program was used to view the optimized molecular structures, including the HOMO and LUMO surfaces (Frisch et al., 2000). The Lee–Yang–Parr expression’s nonlocal correlation; the Vosko–Wilk–Nuair (1980) local correlation functional (III) (B3LYP); and Becke’s three-parameter hybrid exchange functional were all used (Becke, 1993). For carbon and oxygen, the basis set 6-31G (d, p) was used. During the theoretical analysis, the LANL2DZ basis set (Patersson and Al-Latham, 1991) and Hay and Wadt pseudo potentials (Hay and Wadt, 1985; Wadt and Hay, 1985) were employed. The theoretical studies for various quantum chemical parameters were hence carried out employing the Gaussian 09 (Revision B.04) software as previously reported (Dhonchak et al., 2021; Mohmad et al., 2022).

### *In vitro* antibacterial activity

By considering the clinical importance of bacterial pathogens, four bacterial strains were selected to check antibacterial potential of the iridium (III) complex. All the bacterial strains were procured from the Microbial Type Culture Collection (MTCC), Institute of Microbial Technology (IMTECH), Chandigarh, India. The selected strains included two Gram-positive [*Staphylococcus aureus* (MTCC-96) and *Bacillus subtilis* **(**MTCC-121)] and two Gram-negative [*Escherichia coli* **(**MTCC 1652**)** and *Pseudomonas aeruginosa* (MTCC 741)] bacteria. These strains were further preserved on the slants of Nutrient Agar. Antimicrobial activity has been recorded in the form of the zone of inhibition after the completion of the incubation period.

### Agar well diffusion Method

The antibacterial potentials of the Ir (III)-CHMTB complex were detected by the agar well diffusion method (Kamal et al., 2015; Kaur et al., 2016; Kamal et al., 2019). The suspensions of selected bacteria were mixed in sterile saline (0.9% NaCl) by taking 16 h preserved cultures and adjusting to 1.5 × 10^8^ cfu ml^−1^ by referring to the 0.5 McFarland standards. 15–20 ml of Nutrient Agar medium was poured into the Petri plate, which was then swabbed with 100 µL inocula of the bacterial culture and left for 15 min to allow for adsorption over the media. The agar wells with a diameter of 8 mm were created into the seeded agar plates with a sterile cork-borer and loaded with a 100 µL volume of the metal complex with a concentration of 5 mg ml^−1^. All the plates were incubated at 37°C for 24 h to promote bacterial growth. The antibacterial activity of the complex has been evaluated using a zone reader ((Hi Antibiotic Zone Scale)) to measure the zone of growth inhibition (including the well diameter) against the test organisms. A broad-spectrum antibiotic, ciprofloxacin (5 mg ml^−1^), was used as a positive control for bacteria growth inhibition. The complex showing significant antibacterial potential was selected further for minimum inhibitory concentration (MIC) studies.

### Determination of minimum inhibitory concentration of Ir (III)-CHMTB complex

MIC is the lowest concentration of an antimicrobial compound that will inhibit the visible growth of a microorganism after overnight incubation. MIC of the Ir (III)-CHMTB complex against bacterial strains has been tested using a modified agar well diffusion method as mentioned above (Kamal et al., 2015; Kaur et al., 2016; Kamal et al., 2019; Mohmad et al., 2022). In this method, twofold serial dilutions of the complex were prepared by dissolving the complex in dimethylsulfoxide (DMSO) to achieve decreasing concentration ranges of 250–3.91 µg/100 µL. A 100 µL volume of each dilution was introduced into wells (in triplicate) in the agar plates already seeded with 100 µL of standardized inoculum (10^6^ cfu ml^−1^) of the bacterial strains. All seeded plates were incubated aerobically at 37°C and observed for the inhibition zones after 24 h. MIC, taken as the lowest concentration of the complex that completely inhibited the growth of the microbe, was indicated in the form of a clear zone of inhibition. It was recorded for the complex against each bacterial strain. Ciprofloxacin was used as a positive control, whereas DMSO was used as a negative control.

### Antioxidant activity *in vitro*

The antioxidant property of complex and the ligand was studied by Radical Scavenging Activity (RSA) toward DPPH, and the reactions were observed by employing a UV-Vis spectrophotometer (Tabrizi et al., 2019; Lewandowski et al., 2020; Lapasam et al., 2021).

### Free radical scavenging of the compounds using 2,2-diphenyl-1-picrylhydrazyl analysis

DPPH radical scavenging is the most regularly applied assay to the evaluation of the antioxidant activity of chemical compounds. The DPPH (methanolic solution) proffers vivid purple color giving an intense absorption band at 517 nm; the color of the solution changes from deep purple to yellow on interaction with the compound exhibiting antioxidant potential. The DPPH free radical scavenging mechanism resides in the fact that a methanolic DPPH solution is reduced in the presence of an antioxidant. The stock solution of DPPH with a concentration 1.0 mM was prepared by precisely weighing 0.0394 g of DPPH and dissolving the same in 100 ml of methanol. A total of 1,000 μg ml^−1^ stock solution of the complex under study was prepared by dissolving 5 mg of the complex in 5 ml of methanol. The solution acting as a negative control was prepared by taking 5 ml of the DPPH solution in a 25 ml volumetric flask from its stock solution and making up the volume with methanol. A varied range of concentrations (500 μg, 250 μg, 125 μµg, 62.5, and 31.25 μg ml^−1^) of Ir (III)-CHMTB complex were prepared from the stock solution. In an attempt to juxtapose the antioxidant activity of the complex and the ligand (CHMTB), lower dilutions of CHMTB of the same concentrations as those of Ir (III)-CHMTB complex were prepared concomitantly. This was followed by incubating the solutions at 37°C for 30 min in darkness. The same concentrations of the positive control, gallic acid, were prepared simultaneously. Finally, the absorbance of each solution was spectrophotometrically determined at 517 nm against the negative control, and the % free radical scavenging was calculated as per the following equation:

% Free radical scavenging = [(A_negativecontrol_-A_sample_)/A_control_] ×100,where A_negative control_ denotes the absorbance of negative control (which contains all reagents except for the test compounds) and A_sample_ denotes absorbance of the antioxidant compounds (gallic acid, Ir (III)-CHMTB, CHMTB).

## Results and discussion

### Spectrophotometric studies

#### Absorption spectrum

**FIGURE 2:**
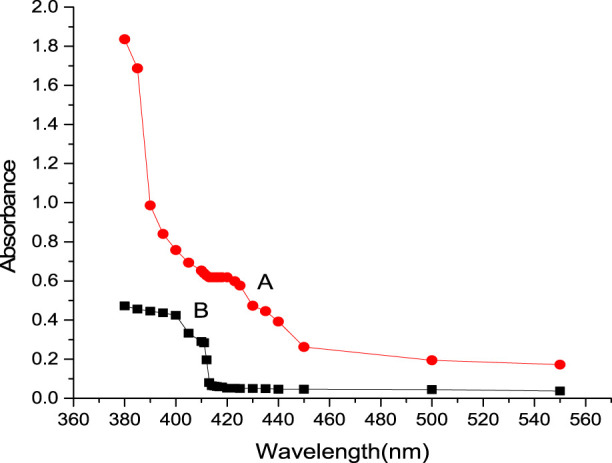
Absorption spectra of Ir (III)-CHMTB complex: spectrum A represents the absorbance of complex against reagent blank; spectrum B represents the absorbance of reagent blank against solvent (pure water).

At a pH of 3.27–4.89, iridium (III) reacted with CHMTB to give an appreciably stable (4 days) yellow-colored compound. The maximum color intensity as exhibited by the complex was in the wavelength range of 413–420 nm when studied against a similarly treated reagent blank, manifesting negligible absorbance in the same visible region (Figure 2). Therefore, all the absorbance measurements were performed at 415 nm.

#### Optimum conditions for Ir (III)-CHMTB complex

None of the solvents, polar or nonpolar, including benzene, toluene, ethyl acetate, cyclohexane, chloroform, methyl isobutyl ketone, isoamyl alcohol, carbon tetrachloride, and dichloromethane, was found effective while evaluating the extraction behavior of Ir (III)-CHMTB complex. In contrast, the complexation tendency of CHMTB under the acidic condition with Ir (III) noticeably attained a maximum in the aqueous phase only and hence was the choice for further investigation.

**FIGURE 3:**
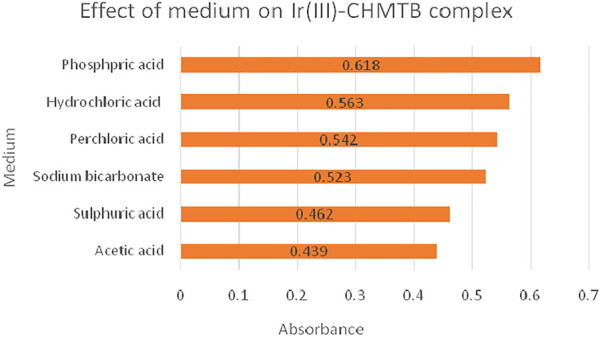
Absorbance of Ir (III)-CHMTB complex in different media.

The formation of the complex was explored in acidic (H_2_SO_4,_ HCl, HClO_4_, H_3_PO_4_, and CH_3_COOH) and basic media (NaHCO_3_, Na_2_CO_3_, and NaOH), resulting in maximum and stable absorbance in the phosphoric acid medium as is shown Figure 3 **.** Highly turbid solutions, however, were obtained while using Na_2_CO_3_ and NaOH as the reaction media.

While studying absorbance diversification of the complex with changing the concentration of H_3_PO_4_, the optical density was observed to be highest at 0.005–0.03M H_3_PO_4_ within the pH range of 3.27–4.89 (Table 1); hence, it was selected as the ideal condition for complexation. Complete complexation was further confirmed by variable addition of the ligand, CHMTB solution to the reaction mixture, and being maximum and constant with 0.8–1.2 ml of its ethanol solution (Table 1); thus, it was fixed as the optimum condition for Ir (III)-CHMTB complex.

**TABLE 1:** Effect of physical variables on the optical density of Ir (III)-CHMTB complex

H_3_PO_4_^x^/M	0.0025	0.005–0.030	0.035	0.040	0.050	0.06	0.075	0.085	
pH	5.11	4.89–3.27	2.96	2.89	2.65	2.57	2.63	1.97	
Absorbance	**0.485**	**0.618**	**0.594**	**0.568**	**0.528**	**0.479**	**0.386**	**0.276**	
CHMTB^y/^ml	0.1	0.3	0.5	0.7	0.8–1.2	1.3	1.5	1.7	2.0
Absorbance	**0.066**	**0.187**	**0.316**	**0.586**	**0.618**	**0.562**	**0.410**	**0.372**	**0.339**

Conditions^x^Ir (III) = 10 μg; 0.1% ethanolic CHMTB = 1 ml; solvent = water; volume of water phase = 10 ml; λ_max_ = 415 nm; pH = variable. ^y^pH = 3.73; the remaining conditions are the same as in (x) excluding reagent concentration. Bold values represent the variable molar concentration of H_3_PO_4_ required as complexing medium.

### Interference studies

To evaluate the tolerance level and selectivity of the Ir (III)-CHMTB system, the impact of analytically important anions or complexing agents and cations was explored (Table 2 and Table 3). Under the ideal set of conditions for the system, the addition of foreign ions was done to Ir (III) (10 µg in 10 ml aqueous volume) initially in pretty large amounts, followed by the addition of small amounts in cases where appreciable interference was observed. None of the complexing agents/anions was found to interfere. However, among 32 selected cations, V(V) showed interference even in trace levels, enhancing the color intensity to a much higher value but was made ineffective in the presence of ascorbic acid acting as the masking agent.

**TABLE 2:** Impact of anions/complexing agents on the Ir (III)-CHMTB complex.

Anion/complexing agent^a^	Salt added	Tolerance level Mg/10 ml	Anion/complexing agent^a^	Salt added	Tolerance level Mg/10 ml
Thiocyanate	NaSCN	100	Carbonate	Na_2_CO_3_	80
EDTA “Disodium”	C_10_H_10_N_2_Na_2_O_8_	100	Phosphate	Na_3_PO_4_	80
Nitrite	NaNO_2_	100	Tartrate	KNaC_4_H_4_O_6_	50
Thiourea	CH_4_N_2_S	100	Dithionite	Na_2_S_2_O_4_	50
Iodide	KI	100	Hydrazinium ion	N_2_H_6_SO_4_	50
Sulfate	Na_2_SO_4_	100	Fluoride	NaF	50
Sulfite	Na_2_SO_3_	100	Oxalate	K_2_C_2_O_4_	50
Chloride	NaCl	100	Acetate	CH_3_COONa	50
Bromide	KBr	100	Ascorbic acid	C_6_H_8_O_6_	50
Nitrate	NaNO_3_	100	Citrate	Na_3_C_6_H_5_O_7_	50
Sulfosalicylic acid	C_7_H_6_O_6_S	100	Glycerol	C_3_H_8_O_3_	1 ml
Bicarbonate	NaHCO_3_	100	Hydrogen peroxide	H_2_O_2_ (100 vol 30%)	1 ml

a Conditions as in the procedure of determination.

**TABLE 3:** Impact of cations on Ir (III)-CHMTB complex.

Cation^*^	Salt added	Tolerance level, Mg/10 ml	Cation^*^	Salt added	Tolerance level, mg/10 ml
Mg (II)	MgCl_2_	10	Fe (II)	FeSO_4_.7H_2_O	1.0
Cr (III)	CrCl_3_	10	Fe (III)	FeCl_3_	1.0
Zn (II)	ZnCl_2_	10	Sn (II)	SnCl_2_	1.0
Co (II)	CoCl_2_.6H_2_O	10	Nb (V)	Nb_2_O_5_	1.0
Ni (II)	NiSO_4_	10	Pd (II)	PdCl_2_	1.0
Sr (II)	SrSO4	10	Ru (III)	RuCl_3_	1.0
Mn (II)	MnCl_2_.4H_2_O	10	Os (VIII)	OsO_4_	1.0
Se (IV)	SeO_2_	10	Au (III)	AuCl_3_	0.8
Hg (II)	HgSO_4_	10	Mo (VI)	(NH_4_)_2_MoO_4_	0.8
Ca (II)	CaCl_2_	10	Zr (IV)	ZrOCl_2_.8H_2_O	0.8
Ag (I)	AgNO_3_	10	As (V)	Na_2_HAsO_4_	0.8
Cu (II)	CuSO_4_	10	Pt (IV)	H_2_PtCl_2_	0.8
Al (III)	AlCl_3_	10	W(VI)	Na_2_WO_4_.2H_2_O	0.8
Cd (II)	CdCl_2_	10	Ti (IV)	TiO_2_	0.5
Ba (II)	BaCl_2_.H_2_O	8	Ce(IV)	NH_3_ [Ce(NO_3_)_6_]	0.5
Pb (II)	PbNO_3_	5	V(V)**	NaVO_3_	0.5
Cr (VI)	K_2_Cr_2_O_7_	5	—	—	—

*Parent oxidation state in parentheses; **with the addition of ascorbic acid (40 mg) as a masking agent.

### Optical and statistical parameters

**FIGURE 4:**
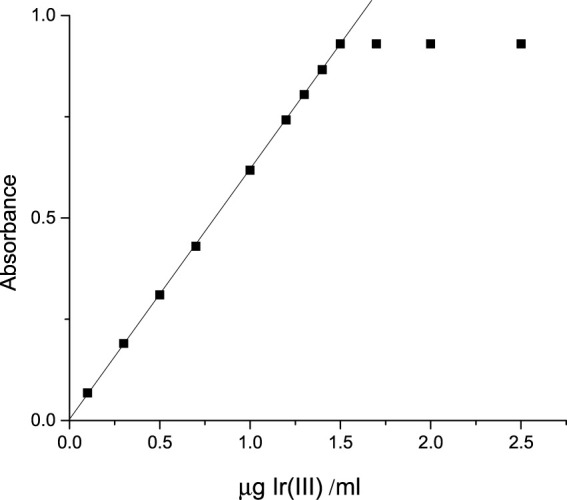
Beer’s law range of Ir (III)-CHMTB.

**FIGURE 5:**
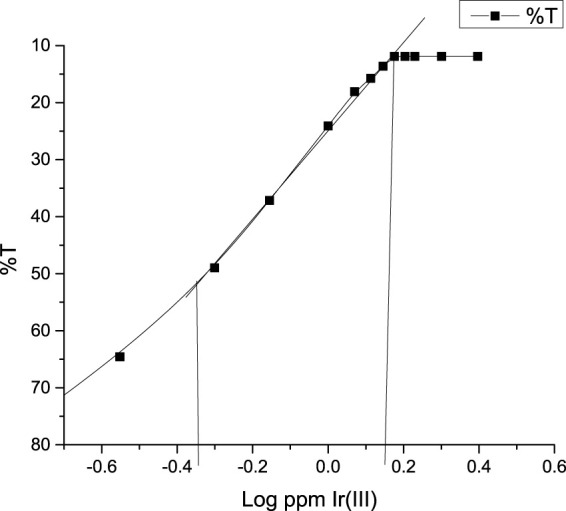
Optimum determination range (Ringbom’s range) of the complex at a wavelength of 415 nm Ir (III)-CHMTB complex.

Under the ambient conditions of the studied system, an illustrious equation for spectrophotometric analysis was obtained from Beer’s law. Ir (III)-CHMTB complex adhered to a linear response between 0.0 and 1.5 µg Ir (III) mL^−1^ (Figure 4), whereas Ringbom’s plot (Ringbom, 1938) exhibited an optimum range of determination as 0.1725–1.4976 ppm of Ir (III) (Figure 5). The molar attenuation coefficient was enormously high amongst the binary complexes studied so far of the metal with a value of 1.188×10^5^ L mol^−1^ cm^−1^ with Sandell’s sensitivity of 0.00162 μg cm^−2^. The linear relationship between variables of the equation of straight line of y = 0.6158 x + 0.0036, absorbance (dependent), and concentration (independent), respectively, is revealed by the coefficient of the determination of the complex with a value of 0.9999. The various optical and statistical parameters are shown in Table 4.

**TABLE 4:** Spectral and qualitative characteristics of the studied complex.

S. No.	Criterion	Stipulations
1	λ_max_	413–420 nm
2	Extinction coefficient	1.188 × 10^5^ L mol^−1^ cm^−1^
3	Sandell’s sensitivity	0.00162 μg cm^−2^
4	Beer’s law range	0–1.5 µg Ir (III) ml^−1^
5	Ringbom’s optimum determination range	0.1725–1.4976
6	Rectilinear regression equation	Y = 0.6158 x + 0.0036
7	Parametric statistic (r)	0.9999
8	Detection limit	0.01193 μg ml^−1^
9	Root mean square deviation	± 0.001095
10	Coefficient of variation	0.1774%
12	Stoichiometry (M:L)	1:2
13	Stability	4 days

#### Composition of the absorbing species

**FIGURE 6:**
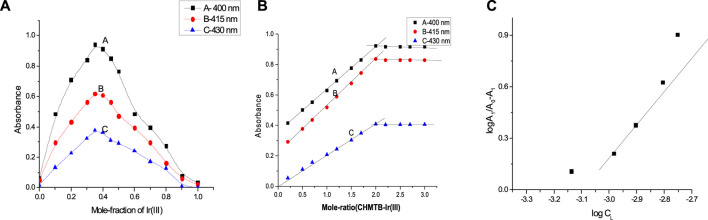
Determination of the composition of the complex **(A)** Job’s continuous variations method, **(B)** mole-ratio method, and **(C)** equilibrium shift method.

For ascertaining the metal-to-ligand ratio and hence the geometry of the formed Ir (III)-CHMTB coordination compound, Job’s continuous variations (Job, 1928; Vosburgh and Cooper, 1941), mole Ratio (Yoe and Jones, 1944), and equilibrium shift methods (Tarasiewicz et al., 1977) were employed (Figure 6). All of these methods deduced and confirmed a metal-to-ligand 1:2 stoichiometry in the studied compound. The slope of 2.12 as obtained from the equilibrium shift method confirms 1:2 M:L composition of the Ir (III)-CHMTB complex.

**FIGURE 7:**
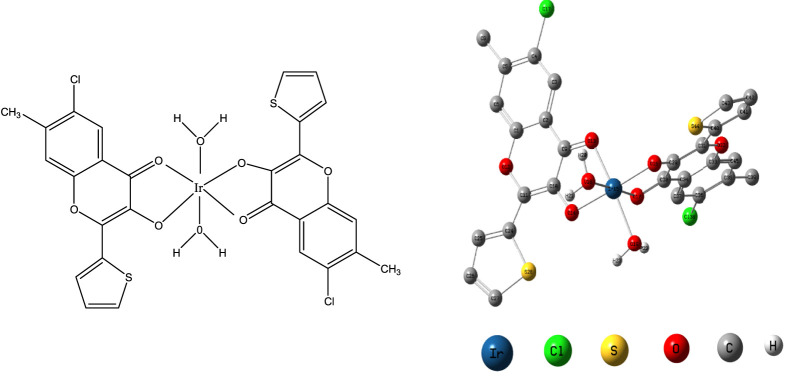
Proposed and optimized structures of the investigated compound.

The experimentation done above led to the following (Figure 7) proposed structure of the studied complex along with its optimized form.

The geometrical properties of the optimized octahedral complex as evaluated by the B3LYP/6-31G (d, p) method are shown in Table 5.

**TABLE 5:** Optimized geometrical variables of the complex [Ir (III)-CHMTB].

Variable	Bond distance (A°)	Variable	Bond angle (°)
Ir-O13	2.011	O13-Ir-O17	98.02
Ir-O14	2.060	O13-Ir-O18	82.61
Ir-O16	2.063	O13-Ir-O19	174.71
Ir-O17	2.031	O13-Ir-O14	82.94
Ir-O18(H_2_O)	2.156	O14-Ir-O16	177.28
Ir-O19(H_2_O)	2.199	O14-Ir-O17	101.07
—	—	O14-Ir-O19	92.38
—	—	O17-Ir-O18	178.99

### Frontier molecular orbital analysis and quantum chemical molecular descriptors

Herein, E_gap_ (HOMO / LUMO energy gap) and the various quantum chemical molecular descriptors are evaluated employing calculations as described earlier (Mohmad et al., 2022) and reported in Table 6. It is obvious from the table that there is a decrease in chemical hardness of ligand by 1.5496 eV after binding to metal, revealing its lower chemical stability and hence more chemical reactivity. According to Table 6, electronegativity (μ), a measure of the tendency of an atom or a group to attract electrons (Geerlings et al., 2003), is higher for the ligand than the complex indicating a high flow of charge density from the reagent to the metal ion. Based on the results, the studied complex is noted to have a higher electrophilicity index (Parr et al., 1999) in comparison to the reagent. This means that the complex has strongly electrophilic, whereas the ligand is nucleophilic in nature. Finally, it can be observed from Table 6 that the estimated electron donating and electron accepting powers of the studied complex are higher than those of the ligand.

**TABLE 6:** Energy gap and quantum chemical molecular descriptors in eV.

Compound	E_HOMO_	E_LUMO_	E_g_	I	A	Η	S	Μ	Ω	ω−	ω+
CHMTB	−6.0934	−2.3282	3.7652	6.0934	2.3282	1.8826	0.5311	4.2108	4.7091	7.0498	2.8390
Ir (III)-CHMTB Complex	−3.1491	−2.4830	0.6661	3.1491	2.4830	0.3330	3.0030	2.8160	11.9066	13.3549	10.5389

**FIGURE 8:**
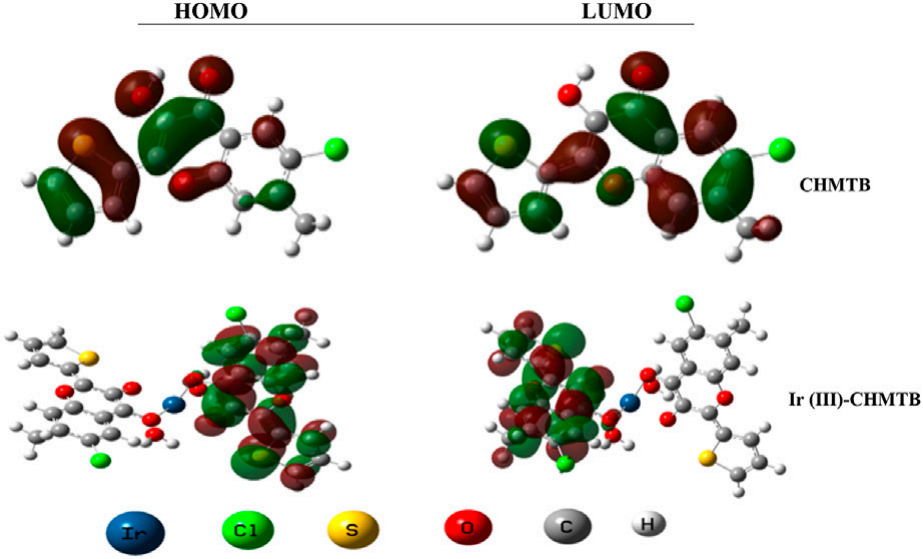
HOMO/LUMO outlines of CHMTB and its Ir (III) complex.

Figure 8 shows the HOMO/LUMO representations of the ligand, CHMTB, and its metal ion complex. The figure clearly denotes that the highest occupied HOMO and lowest unoccupied LUMO are located over the entire molecule of ligand. However, both of them for the complex are mainly contributed by the ligand.

### Analytical, antibacterial, and antioxidant applications

#### Analytical applications

In order to check the versatility and applicability of the method, it was employed in a large number of synthetic mixtures of varying compositions with highly reproducible and satisfactory results, as is indicated from Table 7, by the values of Ir (III) found in various samples, a result of the triplicate analysis. Additionally, the applicability of the method is strengthened by the adequate analysis of iridium in two of its most important alloys, namely, iridosmine and osmiridium (Table 7).

**TABLE 7:** Investigation of miscellaneous mixtures and alloys by the suggested method.

S.No.	Constitution of the matrix^a^	Ir (III) taken	Ir (III) found
		(µg/10 ml)	(µg/10 ml^)^ ^b^
1	Pd (0.1), V* (0.1), Nb (0.1)	10	9.89 ± 0.0244
2	Co (2), Mn (2), Hg (2)	5	4.95 ± 0.0355
3	Pb(2), Ni(3), As (0.5)	10	9.85 ± 0.0540
4	Cu(3), Zn (2), Cr^III^(3),Mg (2)	15	14.91 ± 0.0286
5	Ce (0.5), Fe^III^(0.3),Ag (2), Ni(2)	12	12.01 ± 0.0124
6	Zn (2), Sr (0.5), Mg (2)	7	7.04 ± 0.1110
7	Ru (0.1), Zr (0.1), Ce (0.1)	15	14.90 ± 0.0491
8	Ca (2),Mg (3),W (0.05),Mo (0.1)	5	5.12 ± 0.00814
9	Pt (0.1), Al (5),Ag (2)	8	7.95 ± 0.00798
10	Nb(0.1), Mo (0.1), Zr (0.1)	5	4.93 ± 0.00816
12	Mg (5),Sn(0.5),W (0.05)	5	5.00 ± 0.0163
13	Ni(2), Al (2), Fe^II^(0.1)	7	6.95 ± 0.0648
14	Ba (2), Pd (0.5), As (0.5)	5	4.95 ± 0.0205
15	Os(0.4)^c^	0.6	0.59 ± 0.00186
16	Os(0.8)^c^	0.2	0.19 ± 0.01247

a Mg/10 ml amount of metal ion in parentheses.

b Average of triplicate analysis ± SD.

c Composition analog to iridosmine and osmiridium, respectively. *With the addition of ascorbic acid, 40 mg

#### Evaluation of *in vitro* antibacterial activity

**FIGURE 9:**
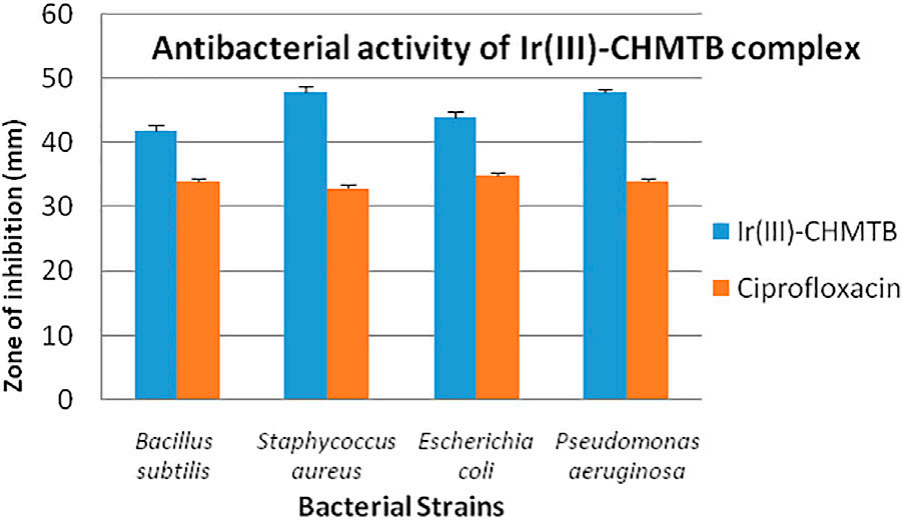
*In vitro* antibacterial activity of Ir (III)-CHMTB complex.

As discussed in the methodology, two Gram-positive [*Bacillus subtilis* (MTCC-121) and *Staphylococcus aureus* **(**MTCC-96)] and Two Gram-negative [*Escherichia coli* (MTCC 1652) and *Pseudomonas aeruginosa* **(**MTCC 741)] bacterial strains were selected to check the antibacterial potential of the newly synthesized Ir (III)-CHMTB complex by determining their MICs. The tested complex showed a highly significant zone of growth inhibition against all the bacterial strains, as mentioned in Table 8 and Figure 9. It inhibited the bacterial cultures in the range of 42–48 mm (zone of inhibition). Ciprofloxacin has been used as the positive control to compare the results. Ciprofloxacin showed a zone of inhibition in the range of 33–35 mm, which was less in comparison to the tested complex.

**TABLE 8:** Antibacterial activity of the complex displaying diameter of growth inhibition in mm.

Solutions	Diameter of growth inhibition (mm)^a^			
	*Bacillus subtilis*	*Staphylococcus aureus*	*Escherichia coli*	*Pseudomonas aeruginosa*
Ir (III)-CHMTB	42 ± 0.86	48 ± 0.75	44 ± 0.86	48 ± 0.45
Ciprofloxacin	34 ± 0.57	33 ± 0.45	35 ± 0.50	34 ± 0.57

a Values, including the diameter of the well (8 mm), are the mean of triplicates ± SD.

**FIGURE 10:**
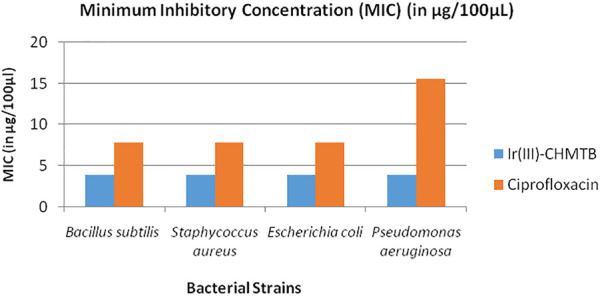
Minimum inhibitory concentration of Ir (III)-CHMTB complex against bacterial strains.

To further compare the antibacterial activities of the complex with the standard antibiotic, the MIC values of both were compared as depicted in Table 9 and Figure 10. The antibacterial effects against the tested strains were found more significant for the complex, which showed MIC values up to 3.91 μg/100 μL. As compared to the complex, ciprofloxacin showed MIC values in the range of 7.81–15.62 μg/100 μL. It is noteworthy that the Ir (III)-CHMTB complex was highly active against the tested bacterial strains, and also, the complex showed activity higher than that exhibited by ciprofloxacin.

**TABLE 9:** Minimum inhibitory concentration (MIC) (in μg/100 μL) of Ir (III)-CHMTB complex.

Solutions	Gram-positive bacteria		Gram-negative bacteria	
	*Bacillus subtilis*	*Staphylococcus aureus*	*Escherichia coli*	*Pseudomonas aeruginosa*
Ir (III)-CHMTB	3.91	3.91	3.91	3.91
Ciprofloxacin	7.81	7.81	7.81	15.62

#### DPPH radical scavenging activity

The free radicals generated by various metabolic processes or UV radiations cause the deterioration of DNA, proteins, and lipids. Under such circumstances, the administration of antioxidants from outside play a vital role for the protection of biological systems. Antioxidants work by scavenging free radicals and inhibiting chain reactions by giving the free radical an electron or hydrogen. As a result, there has been a surge in interest in creating new, safer antioxidants with fewer adverse effects.

**FIGURE 11:**
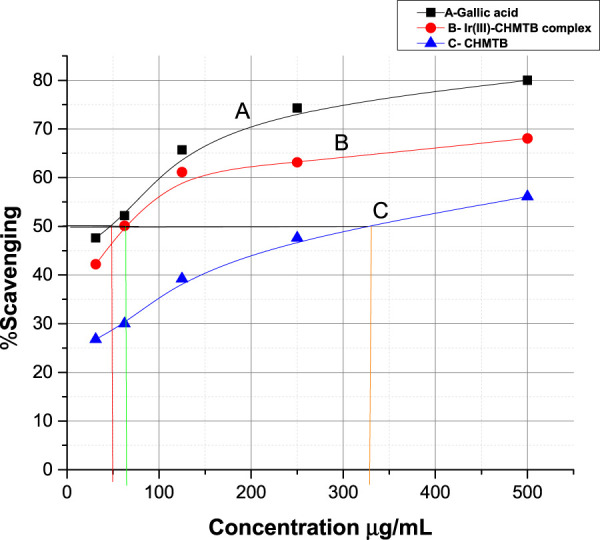
Percent radical scavenging activity of (A) gallic acid, (B) Ir (III)-CHMTB complex, and (C**)** CHMTB.

**FIGURE 12:**
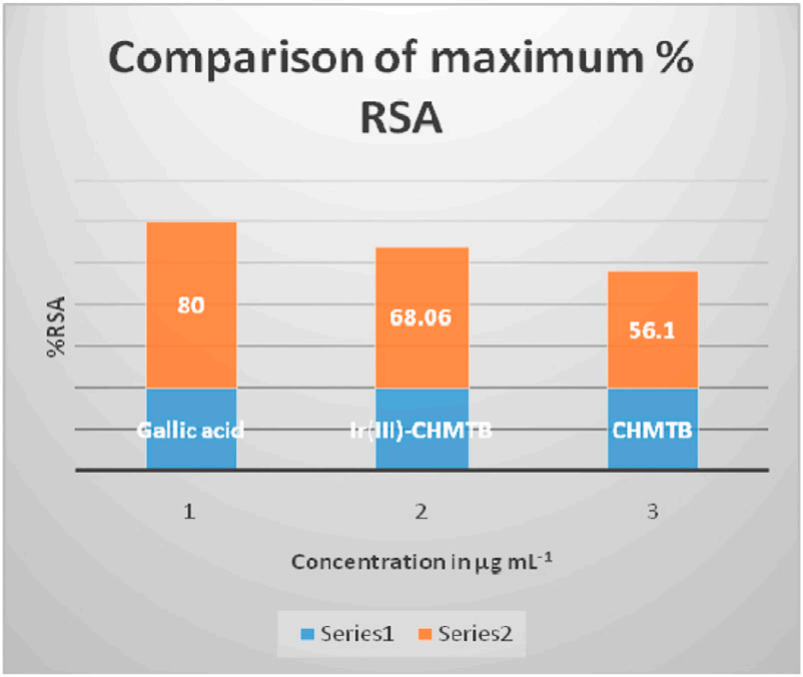
Comparison of percent radical scavenging activity of the selected compounds.

The antioxidant activity at various concentrations of CHMTB and Ir (III) complex was determined by measuring the de-colorization of DPPH^.^. The RSA increased with the increase in the concentration of the compounds. CHMTB and Ir (III)-CHMTB complex exhibited the highest scavenging activity of 56.1% and 68.06%, respectively, at 500 μg ml^−1^ concentration. Under experimental conditions, IC_50_ of Ir (III) complex is 62.5 μg ml^−1^. The RSA can be compared in the following order: gallic acid > Ir (III)-CHMTB complex > CHMTB (Table 10; Figure 11, Figure 12). Thus, the Ir (III)-CHMTB complex displays greater scavenging activity than the free ligand and, hence, can be used as an effective antioxidant.

**TABLE 10:** Antioxidant activity of the studied ligand and its complex in terms of % scavenging activity.

Concentration	% scavenging		
µg ml^−1^	CHMTB*	Ir (III)-CHMTB**	Gallic acid***
31.25	26.8	42.19	47.6
62.5	30	50.0	52.2
125	39.2	61.11	65.7
250	47.6	63.14	74.3
500	56.1	68.06	80

IC_50_ values = *350 µg ml^−1^, **62.5 µg ml^−1^, and***50 µg ml^−1^.

## Conclusion

In summary, in the presented work, for the first time, CHMTB was utilized successfully for the microgram level analysis of iridium (III) employing the spectrophotometric determination technique. Iridium (III) instantaneously reacts with CHMTB in the phosphoric acid medium at an ambient pH of 3.73 to form a stable yellow-colored non-extractable complex cohering to a linear response up to 1.5 µg Ir (III) mL^−1^. The accuracy and reproducibility of the method are confirmed by the corresponding values of relative standard deviation (RSD) and the coefficient of variance as 0.1774% and 0.9999, respectively. The stoichiometric constitution of the developed complex, as validated by the studied methods, was 1:2 (M:L). Recently, several binary complexes of Ir (III) have been investigated and studied spectrophotometrically. However, in addition to being simple, versatile, and economic, the current method is searched to be highly sensitive in comparison to the previously studied binary complexes of Ir (III) (Kuchekar et al., 2014; Borgave and Barhate, 2016; Kuchekar et al., 2019) as can be inferred from the molar attenuation coefficient of 1.188×10^5^ L mol^−1^ cm^−1^. DFT studies were employed to get know-how about structural details and electronic properties of the complex. The DFT simulations not only revealed an energy-optimized structure for CHMTB and its complex with Ir (III), but also confirmed the ligand and complex to have significant nucleophilic and electrophilic properties, respectively. By considering the therapeutic applications of iridium and its complexes, the Ir (III)-CHMTB complex was screened for antioxidant and antibacterial activities following the standard protocols of determination of the same. Successful elucidation of the antioxidant and antibacterial activities made the studied complex serve as a limelight for the formation of antioxidant agents and bactericidal drugs, a novel addition to the iridium-based therapeutic agents. Additionally, with reference to responsiveness, speed, and versatility, the current study aptly contemplates the already reported methods (Druskovic and Vojkovic, 2003; Li-Ming et al., 2008; Kuchekar et al., 2014; Borgave and Barhate, 2016; Kuchekar et al., 2019; Mohmad et al., 2022).

## Data Availability

The original contributions presented in the study are included in the article/Supplementary Material. Further inquiries can be directed to the corresponding authors.
