# Corrigendum: Radical scavenging capacity, antibacterial activity, and quantum chemical aspects of the spectrophotometrically investigated iridium (III) complex with benzopyran derivative

**DOI:** 10.3389/fphar.2022.1059892

**Published:** 2022-10-24

**Authors:** Masrat Mohmad, Nivedita Agnihotri, Vikas Kumar, Mohammad Azam, Saikh Mohammad Wabaidur, Raj Kamal, Rakesh Kumar, Mahboob Alam, Sadegh Kaviani

**Affiliations:** ^1^ Department of Chemistry, Maharishi Markandeshwar (Deemed to Be University), Ambala, India; ^2^ Department of Biotechnology, Maharishi Markandeshwar (Deemed to Be University), Ambala, India; ^3^ Department of Chemistry, College of Sciences, King Saud University, Riyadh, Saudi Arabia; ^4^ Department of Chemistry, Kurukshetra University, Kurukshetra, India; ^5^ Department of Chemistry, MCM DAV College, Kangra, Himachal Pradesh, India; ^6^ Department of Safety Engineering, Dongguk University, Gyeongju, South Korea; ^7^ Department of Physics, Kazan Federal University, Kazan, Russia

**Keywords:** iridium, quantum chemical studies, spectrophotometric determination, antibacterial, antioxidant

In the published article, there was an error in affiliation 7. Instead of “Department of Chemistry, Faculty of Science, Research Centre for Modelling and Computational Sciences, Ferdowsi University of Mashhad, Mashhad, Iran”, it should be “Department of Physics, Kazan Federal University, Kazan, Russia”.

The authors apologize for this error and state that this does not change the scientific conclusions of the article in any way. The original article has been updated.

